# Born without a Silver Spoon: A Review of the Causes and Consequences of Adversity during Early Life

**DOI:** 10.1093/icb/icad061

**Published:** 2023-06-06

**Authors:** Mark C Mainwaring, Bret W Tobalske, Ian R Hartley

**Affiliations:** School of Natural Sciences, Bangor University, Bangor LL57 2DG, UK; Field Research Station at Fort Missoula, Division of Biological Sciences, University of Montana, Missoula, MT, USA; Field Research Station at Fort Missoula, Division of Biological Sciences, University of Montana, Missoula, MT, USA; Lancaster Environment Centre, Lancaster University, Lancaster LA1 4YQ, UK

## Abstract

A huge amount of research attention has focused on the evolution of life histories, but most research focuses on dominant individuals that acquire a disproportionate level of reproductive success, while the life histories and reproductive tactics of subordinate individuals have received less attention. Here, we review the links between early life adversity and performance during adulthood in birds, and highlight instances in which subordinate individuals outperform dominant conspecifics. Subordinate individuals are those from broods raised under high risk of predation, with low availability of food, and/or with many parasites. Meanwhile, the broods of many species hatch or are born asynchronously and mitigation of the asynchrony is generally lacking from variation in maternal effects such as egg size and hormone deposition or genetic effects such as offspring sex or parentage. Subordinate individuals employ patterns of differential growth to attempt to mitigate the adversity they experience during early life, yet they overwhelmingly fail to overcome their initial handicap. In terms of surviving through to adulthood, subordinate individuals employ other “suboptimal” tactics, such as adaptively timing foraging behaviors to avoid dominant individuals. During adulthood, meanwhile, subordinate individuals rely on “suboptimal” tactics, such as adaptive dispersal behaviors and competing for partners at optimal times, because they represent the best options available to them to acquire copulations whenever possible. We conclude that there is a gap in knowledge for direct links between early life adversity and subordination during adulthood, meaning that further research should test for links. There are instances, however, where subordinate individuals employ “suboptimal” tactics that allow them to outperform dominant conspecifics during adulthood.

## Introduction

A huge amount of research attention has focused on the evolution of life histories in a diverse range of vertebrate and invertebrate taxa (Roff 1992; Stearns 1992). More specifically, a plethora of studies have focused on the acquisition of reproductive success by individuals that thereby pass on their genes to future generations. Such studies often refer to concepts such as the lifetime reproductive success of individuals (Clutton-Brock 1988; Newton 1989) and to the silver-spoon effect, whereby individuals raised under advantageous conditions consequently become dominant adults and so acquire relatively high levels of reproductive success (Lindström 1999; van de Pol et al. 2006).

Most studies focus on the acquisition of reproductive success by individuals that are successful in passing on their genes to future generations, so our understanding tends to be biased toward life histories and reproductive tactics of dominant individuals (Clutton-Brock 1988; Andersson 1994). For example, Eurasian oystercatchers (*Haematopus ostralegus*) (van de Pol et al. 2006) and black-capped chickadees (*Poecile atricapillus*) (van Oort and Otter 2005), raised in high-quality habitats with an abundance of prey, subsequently also settled and bred in high-quality habitats. Meanwhile, in spotted hyenas (*Crocuta crocuta*) that live in matriarchal clans, places within social hierarchies are inherited vertically so that the daughters of dominant females are dominant over females raised in less dominant family lines (Ilany et al. 2021). Additionally, high-quality juveniles that have high body mass and condition tended to have the highest reproductive success as adults in great tits (*Parus major*) (Tilgar et al. 2010) and wild boar (*Sus scrofa*) (Vetter et al. 2016).

So, while there is evidence from a range of taxa that a good start in life can lead to relatively high success as an adult, the outcomes for individuals that have had a disadvantaged beginning have been largely overlooked, even though they should also be expected to maximize their own reproductive success. Research examining links between adversity experienced in early life and performance in adulthood has focused extensively on the trade-off between growth and reproductive success (Metcalfe and Monaghan 2001). The “grow now, pay later” concept proposes that individuals might invest in relatively rapid growth during early development, for example, to better compete against siblings (Royle et al. 1999) or to reduce predation risk, but then subsequently pay for that investment later in their lives by having shorter lifespans, fewer reproductive opportunities, and thus relatively lower lifetime reproductive success (Richner et al. 1989; Lindström 1999; Metcalfe and Monaghan 2001; Fitze et al. 2004; Fisher et al. 2006; Auer et al. 2010; Krause and Naguib 2011; Mainwaring and Hartley 2012; Spagopoulou et al. 2020).

Research on birds provides a large proportion of the studies relevant to our understanding of early life impacts on fitness, probably because they are relatively easy to mark, monitor, and manipulate for key characteristics such as egg number, reproductive success, and survival. Here, we thus focus on birds and begin by reviewing the sources of the variation in the quality of individuals in their early life period before outlining factors that might mitigate against a poor start in life. We explore links between conditions experienced during early life and performance during adulthood, and highlight those instances where, perhaps counterintuitively, individuals that experience adversity have a higher relative fitness in later life.

## Sources of variation in offspring traits

Avian offspring are rarely born equal and, indeed, there can be considerable variation in the genotype or phenotype of individuals from early in their development up to the point at which they become independent of their parents, and this variation can impact later life in a variety of ways. Perhaps the most obvious variation can be seen directly within broods, where there are often one or two offspring that are smaller than the rest, usually because they are born later within asynchronously hatched broods and are unable to compete as successfully as their siblings for parentally provisioned food. However, for iteroparus species, like most bird species, there can be variation between individual offspring between broods, for example, first or second broods within a single season, or between nests in different seasons due to changes in parental age or annual variation in weather or food supplies. Finally, there can be variation in offspring between broods of different breeders due to parental differences. Factors such as parentage and offspring sex might also introduce variation or interactions with the environment ,which impact ofitness.

## Within-brood variation

Variation in offspring quality within avian broods can arise due to a range of variables, many of which are under the control of one or both parents. These include incubation and consequent hatching regimes, allocation of resources, such as hormones or proteins to embryos, and in some species, sex allocation or control of parentage through sperm competition.

### Hatching asynchrony

The primary cause of offspring being subordinate within nests is hatching asynchrony, whereby offspring from a single reproductive event hatch over an extended period such as several days. While the subordinates are waiting to hatch, their siblings gain a head start in growth, and gain mass or size, which impacts upon scramble competition for parentally provided food (Mock and Parker 1997). In birds, hatching asynchrony is a maternal or post-hatching environmental effect determined by parental decisions regarding the onset of incubation, and while most studies examining the causes and consequences of hatching asynchrony have involved birds (Magrath 1990; Stoleson and Beissinger 1995), hatching asynchrony is taxonomically widespread and occurs in sharks (Gilmore 1993), amphibians (Ryan and Plague 2004), reptiles (While and Wapstra 2009), and insects (Smiseth and Morgan 2009).

Female birds are physiologically constrained to lay a maximum of one egg per day yet embryonic development does not begin until incubation starts. While some groups, such as owls and gulls, commence incubation when the first egg is laid, other species, particularly passerines, tend to begin incubation when the penultimate egg is laid; both strategies lead to asynchronous hatching of eggs and a hierarchy of size within the brood (Mock and Forbes 1995; Stoleson and Beissinger 1995). The earliest hatched offspring are often referred to as “core” offspring, and exhibit low variation in survival and growth rates, while the later hatched offspring are often referred to as “marginal” offspring and commonly suffer lower rates of survival and growth than their siblings (Glassey and Forbes 2002; Forbes 2011).

### Egg size and composition

Female parents can mitigate the effects of hatching asynchrony by laying increasingly large eggs as the laying sequence progresses because higher-quality offspring emerge from larger eggs (Krist 2011). There is widespread evidence in birds that egg size increases throughout the laying order so that egg size is higher in later-laid eggs than in early laid eggs (Slagsvold et al. 1984; Williams 1994; Krist et al. 2004; Rosivall et al. 2005). For example, Krist et al. (2004) showed that the last-laid eggs in collared flycatcher (*Ficedula albicollis*) broods were 12% larger than the earliest-laid eggs.

Rosivall et al. (2005) also showed that female collared flycatchers laid larger eggs toward the end of the laying sequence, which may mitigate the effects of the size hierarchies created by hatching asynchrony. However, nestlings that hatched from later-laid eggs were lighter and had shorter feathers than nestlings that hatched from earlier-hatched eggs, which was disadvantageous because they are less likely to recruit into the breeding population in the future and also because they are less likely to escape maneuverable predators such as hawks (Rosivall et al. 2005). This indicates that the nestlings raised in later laid, yet larger eggs, were destined to be subordinate to nestlings raised in earlier-laid eggs, reflecting a more general pattern of egg size variation within clutches, which suggests that increasing egg size does not mitigate entirely the effect of hatching asynchrony (Magrath 1990; Mainwaring and Hartley 2012).

Female birds can also influence nestling fitness via the differential investment of egg constituents, such as antioxidants, androgens, and corticosterone through the laying sequence, independent of egg size (Krist 2011). In several species, females deposit more egg constituents throughout the laying sequence so that later-laid eggs receive slightly more hormones than earlier-laid eggs (Schwabl 1996; Lipar et al. 1999; Royle et al., 2001, 2003). However, an experiment in which the eggs of blue tits (*Cyanistes caeruleus*) that were laid first were experimentally made to hatch last showed that variation in the deposition of hormones or egg sizes across the laying order did not influence offspring phenotypes (Mainwaring et al. 2010a).

Most studies show that even though parents do increase the size of later-laid eggs and invest more hormones into those eggs, they do not overcome the size differences created by hatching asynchrony (Schwabl 1996). Further, avian parents usually feed the largest offspring within their broods (Royle et al. 2002a; Mainwaring et al. 2011b; Glassey and Forbes 2002; Draganoiu et al. 2005; Dickens and Hartley 2007; Kilner and Hinde 2008), presumably because they are investing in the largest offspring that are most likely to pass on their genes to future generations. Such favoritism means that earliest hatched offspring reach developmental landmarks—such as acquiring sight and gaining hardened keratin on their lower legs—sooner than their smaller siblings and as they can initiate begging displays sooner and reach higher within nests (Smith and Montgomerie 1991; Teather 1992; Leonard and Horn 1996; Dearborn 1998), they usually win contests for parentally provided food.

### Offspring sex

In most species, males and females differ not only in appearance but also in their life histories, with the two sexes usually varying in key traits such as survival and reproductive success. The impact of sexual selection can lead to sexual dimorphism in size, color, and behaviors, which might themselves be influenced by an individual’s condition as a juvenile. In most bird species, male offspring are larger than females (Mainwaring et al. 2011a; Hartley et al. 2000; Hartley 2007; Kalmbach and Benito 2007). However, raptors such as Eurasian sparrowhawks (*Accipiter nisus*), exhibit reversed sexual size dimorphism so that female offspring are larger than males (Moss 1979). Size differences can directly impact offspring fitness as some parents preferentially provision offspring of one sex (Lessells 2002a, 2002b; Mainwaring et al. 2011b; Kalmbach and Benito 2007). Further, the sex of offspring may vary across the laying order, with males usually hatching from later-laid eggs (Cichoń et al. 2003).

### Offspring parentage

Our understanding of parentage in socially monogamous birds has been revolutionized in the past few decades because of the emergence of molecular genetic tools that facilitate the identification of the genetic parents of offspring. The previous view of monogamous pair bonds, which was one of faithful, true monogamy, has since changed because extra-pair paternity is seen in nearly 90% of bird species (Griffith et al. 2002). Mixed paternity within broods means that some offspring are half-siblings and competition for food increases as the average relatedness of siblings within litters or broods decreases (Briskie et al. 1994; Boncoraglio et al. 2009). Extra-pair offspring are usually larger than within-pair offspring within the same broods, and while this was usually attributed to genetic effects as proposed by the “good genes” hypothesis (Griffith et al. 2002), more recent studies show that extra-pair offspring hatch from earlier laid and thus earlier hatched eggs. Extra-pair offspring are, therefore, generally larger because of the size hierarchies created by hatching asynchrony (Magrath et al. 2009; Ferree et al. 2010). Further, parents neither recognize nor preferentially provision extra-pair offspring within broods (Leonard et al. 1995; Whittingham and Lifjeld 1995; Kempenaers and Sheldon 1996), thereby suggesting that extra-pair nestlings are larger than within-pair offspring due to hatching asynchrony.

## Variation between breeding events

Variation in the quality of offspring, which impacts on their fitness, can vary between, as well as within, breeding events, even when the same parents are involved. This variation may occur, for example, due to differences between first and second broods, differences due to parents getting older or more experienced, or due to parents changing partners, all of which may vary between breeding events or seasons.

Many bird species can have two or more successful breeding attempts in one season and when prey availability or predation risk is not constant (Morrison et al. 2019), there may be differences in reproductive output and the quality of offspring between first and subsequent broods. For example, nestlings from second broods of barn swallows (*Hirundo rustica*) (Banbura and Zielinski 1998) and rock pipits (*Anthus petrosus*) (Askenmo and Unger 1986) are lighter at pre-fledging, and thus, at a disadvantage compared to nestlings from first broods because they are less likely to be recruited into the breeding population (Both et al. 1999).

The breeding success of animals tends to increase with the age, at least partly because parents acquire experience of how to breed optimally over time. In birds, younger parents often raise smaller, and thus disadvantaged offspring than older parents (Sæther 1990; Black and Owen 1995). Bogdanova et al. (2006) examined this relationship and found that younger herring gull (*Larus argentatus*) females laid smaller eggs with reduced amounts of albumen in them, which resulted in those offspring being smaller and competitively inferior to offspring hatching from eggs laid by older females. Older parents, meanwhile, close to the end of their lives sometimes have lower levels of reproductive success than middle-aged parents (Bonamour et al. 2020). This is at least partly because raising offspring increases parental aging, with Landsem et al. (2023) showing that Atlantic puffin (*Fratercula arctica*) parents exhibited increased rates of senescence after raising offspring.

In species with biparental care, breeders sometimes switch partners between breeding events, either through divorce or as a consequence of mortality (Choudhury 1995; Otter and Ratcliffe 1996; Culina et al. 2015). A meta-analysis of 64 species of socially monogamous birds showed that offspring raised by parents prior to divorce are of lower quality, and thus disadvantaged, than offspring raised by parents after divorce (Culina et al. 2015). Otter and Ratcliffe (1996) showed that female black-capped chickadees divorced males in favor of males with high social ranks and subsequently raised better quality offspring, although some studies show that switching partners is not beneficial (Leach et al. 2020).

Although the majority of bird species are monogamous or socially monogamous, some species are polygynous, whereby one male pairs with two or more females. Males care for the females unequally with the primary females receiving the majority of help, while secondary females receive less help (Gibbs et al. 1990; Eens and Pinxten 1994). This means that the total amount of food provisioned to broods of secondary females is less than broods of primary females. As those offspring are lighter at fledging (Pinxten and Eens 1990), they are disadvantaged by being subordinate to the offspring of primary females, which are heavier and thus dominant in intraspecific contests for resources such as food.

Other bird species are cooperative breeders, whereby offspring are cared for by parents and by additional group members known as helpers (Cooper et al. 2020). Nestlings raised in broods where helpers are present are heavier and thus at an advantage over broods raised without helpers (Bolopo et al. 2019; Cooper et al. 2020). Illustratively, Hatchwell et al. (2004) showed that in cooperatively breeding long-tailed tits (*Aegithalos caudatus*), helpers increase the total provisioning rate and thus produced heavier nestlings than broods without helpers. Further, nestlings raised in broods with helpers were more likely to be recruited into the study population than nestlings raised in broods without helpers (Hatchwell et al. 2004), thus demonstrating that offspring raised without helpers are disadvantaged in terms of their likelihood of being recruited into the breeding population.

The size of offspring also varies among years as a result of, for example, weather conditions influencing prey availability, independent of parent quality. In birds, for example, between-year variation in rainfall influences the pre-fledging mass of tree swallows (*Tachycineta bicolor*) (Cox et al. 2019), and among-year variation in temperature influences the pre-fledging mass of birds via changes in the availability of their prey (Emlen et al. 1991; van de Ven et al. 2020).

## Variation between breeders

### Predation risk

Broods raised in locations under a high risk of predation are smaller than conspecifics, because of reductions in parental provisioning rates (Scheuerlein and Gwinner 2006). Yellowhammer (*Emberiza citrinella*) parents reduced their provisioning rates to nests in areas with many predatory corvids, which resulted in nestlings being lighter and thus subordinate to control nestlings raised in areas with few predatory corvids (Dunn et al. 2010). Meanwhile, Coslovsky and Richner (2011) showed that great tits exposed to taxidermy mounts of predatory Eurasian sparrowhawks before and during ovulation went on to raise smaller nestlings than pairs exposed to taxidermy mounts of non-predatory song thrushes (*Turdus philomelos*), even though nestlings were cross-fostered between treatments. The nestlings of mothers breeding in locations of increased predators were therefore smaller in mass, but had longer wings, perhaps enhancing flight performance, than conspecifics raised under the control treatment because the mothers altered either the composition of their eggs or their incubation behavior.

### Food availability

The low availability of quality food can also subject broods or litters to adversity during early life. For example, male swordtail fishes (*Xiphophorus helleri*) exposed to a relatively short spell of restricted food availability during early life ultimately acquired the same body mass as males reared under conditions of *ad libitum* quantities of food, but were subordinate to size-matched individuals during adulthood (Royle et al. 2005). In birds, meanwhile, several studies show that when brood sizes are experimentally increased, to indirectly mimic a situation whereby food is in limited supply because parents provide less food per offspring, those offspring were lighter and thus subordinate to control offspring raised in broods of unchanged sizes (Nilsson and Gårdmark 2001; Gil et al. 2008; Miller 2010).

### Brood parasites

Some bird species are targeted by brood parasites such as cuckoos (*Cuculidae*) and cowbirds (*Molothrus* spp.), where the parents of the host species raise the brood parasitic nestling. While cuckoo nestlings evict host offspring from nests, cowbird offspring share the nest with the host offspring (Davies 2000), meaning that host nestlings raised in nests with brood parasitic nestlings should receive less parentally provided food, and therefore be disadvantaged when compared to nestlings raised in the absence of brood parasitic nestlings because they are smaller and lighter at pre-fledging. Support for that idea comes from studies showing that sharing the nest with brood parasitic brown-headed cowbirds (*Molothrus ater*) caused prothonotary warbler (*Protonotaria citrea*) nestlings to be smaller than nestlings raised in nests without cowbirds (Scharf et al. 2022). Further, the nestlings of nine different host species were smaller and thus disadvantaged when compared to host nestlings raised without cowbird nestlings (Jones et al. 2023).

### Ectoparasite abundance

The abundance of ectoparasites in nests influences the quality of offspring because they feed on the blood of offspring. Studies of great tits in which experimental nests had hen fleas (*Ceratophyllus gallinae*) added to them resulted in offspring being smaller and lighter, and thus subordinate, to offspring raised in nests in which hen fleas were experimentally eliminated (Richner et al. 1993; Oppliger et al. 1994; Tschirren et al. 2003). Also, experimentally increased numbers of louse flies (*Ornithomyia biloba*) in barn swallow nests led to nestlings being smaller and thus subordinate to conspecifics in nests with fewer flies (Saino et al. 1998). However, there was no effect in alpine swift (*Apus melba*) nestlings raised in nests where blood-sucking louse flies (*Crataerina melbae*) were added to nests (Bize et al. 2004).

### Parental body size and competitive ability

The size or competitive ability of parents usually influences the competitiveness of their offspring, with dominant and aggressive parents often raising offspring with similar traits. In monkeys, offspring raised by high-ranking females often win playful interactions with other offspring because their mothers intervene and help them outcompete other offspring (Holekamp and Smale 1991). In birds, meanwhile, experimental pairs of western bluebirds (*Sialia mexicana*) that were exposed to increased competition for nest sites subsequently hatched offspring that grew more rapidly and were larger at pre-fledging than the offspring of control pairs (Hernández et al. 2022).

The personality of parents also influences the size of offspring, with among-individual variation in the aggression of great tit mothers being indirectly linked to the mass of fledglings through its association with female provisioning rates in one of two study years (Thys et al. 2021). Kontiainen et al. (2009) showed that submissive ural owl (*Strix uralensis*) females recruited significantly fewer offspring into the breeding population as they defended their offspring less fiercely than more aggressive conspecifics. Meanwhile, the white-throated sparrow (*Zonotrichia albicollis*) has a genetic mutation that caused it to flip a section of their chromosome so that there are four genotypes and fewer mating opportunities (Tuttle et al. 2016).

### Territory and/or partner quality

The mass of offspring is a strong determinant of dominance, with heavier offspring being dominant over lighter conspecifics. The mass of offspring is sometimes strongly determined by the quality of territory in which they are raised. For example, great tit offspring are provisioned with caterpillars and so as the number of caterpillars is positively correlated with the number of mature oak trees, then high-quality territories contain many mature oak trees (Wilkin et al. 2009). Accordingly, Bell et al. (2014) showed that the mass of blue tit nestlings at pre-fledging was positively correlated with the number of mature oak trees in their territories.

In addition to the availability of food, partner quality also influences offspring fitness. For example, Arct et al. (2019) showed that the genetic similarity between blue tit parents negatively impacted the immune responses of offspring, thus making them more susceptible to diseases than offspring raised by genetically different parents. Bowers et al. (2017) further showed that the quality of house wren (*Troglodytes aedon*) offspring was influenced by the age of a focal parent’s partner with offspring fitness being greater when their parents had a smaller gap in ages than when parents had a larger gap in ages.

## Consequences of variation in juvenile traits

Having outlined the sources of variation in juvenile traits above, we now outline the consequences of such variation for subordinate offspring. More specifically, we outline the ways in which subordinate offspring attempt to mitigate the adverse circumstances they experience during early life both between and within broods. We also examine the links between variation in juvenile traits and their subsequent performance during adulthood.

### Between broods

Subordinate offspring can develop those morphological traits other than mass, such as wings, to maximize fitness as the acquisition of fully developed wings enables them to fledge the nests and thus escape the adverse conditions in which they are raised (Mainwaring and Hartley 2012). Proportionally longer wings for a given body mass likely reduce the energetic cost of flight and facilitates rapid take-off (Norberg 1990). Illustratively, Coslovsky and Richner (2011) showed that great tit nestlings raised in areas under high levels of perceived predation risk were smaller and lighter than offspring in control nests, yet they grew their flight feathers faster. This was interpreted to mean that the offspring raised in areas under high levels of predation risk could fledge sooner and thus escape the area of high predation risk (Coslovsky and Richner 2011).

Food availability also causes variation in offspring phenotypes (Nilsson and Gårdmark 2001). Several studies of birds have increased brood sizes, which mimics a decrease in the availability of food per offspring because parents do not increase their provisioning rates after the experimental enlargement of their broods. Gil et al. (2008) showed that spotless starling (*Sturnus unicolor*) offspring in experimentally enlarged broods were smaller but had larger gapes than control offspring, to compete for parentally provided food. Meanwhile, both mourning doves (*Zenaida macroura*) (Miller 2010) and marsh tits (*Poecile palustris*) (Nilsson and Gårdmark 2001) raised in enlarged broods were also lighter but grew their flight feathers significantly faster, than control offspring to rapidly escape the adverse conditions they experienced in the nest.

Nestling birds also experience adverse natal conditions when their nests contain ectoparasites. The experimental addition of ectoparasites to nests results in offspring being smaller and lighter than control nestlings (Richner et al. 1993; Tschirren et al. 2003). Further, barn swallow nestlings raised in nests in which the number of louse flies was experimentally increased grew smaller and lighter, but had longer wings, than nestlings raised in control nests. The investment of resources in wing growth was thought to enable the nestlings to fledge as soon as possible and escape the adverse conditions within nests that contained large numbers of ectoparasites (Saino et al. 1998).

In addition to ectoparasites, some bird species are targeted by brood parasitic bird species such as cowbirds and cuckoos, who lay eggs in the nests of hosts so that the hosts raise the parasite’s offspring. The offspring of the brood parasites are completely unrelated to the offspring of the hosts and so competition between host and parasitic offspring is not constrained by indirect genetic fitness benefits (Kilner et al. 1999; Davies 2000). Ortega and Cruz (1992) examined patterns of differential growth in brood parasitic brown-headed cowbird nestlings, which are lighter and smaller than the nestlings of their yellow-headed blackbird (*Xanthocephalus xanthocephalus*) hosts. The brown-headed cowbird nestlings were lighter than yellow-headed blackbird nestlings, and while the yellow-headed blackbird nestlings had larger gapes *per se*, the brown-headed cowbird nestlings developed significantly larger gapes relative to their weight, meaning that they invested more resources in gape development than their hosts (Ortega and Cruz 1992).

### Within broods

The size hierarchies created by hatching asynchrony result in late hatched avian nestlings becoming subordinate to early hatched nestlings (Clark and Wilson 1981; Forbes 2011). Although late hatched, subordinate offspring beg more intensely for parentally provided food, they usually receive less food than their earlier-hatched siblings (Mainwaring et al. 2010b, 2011b; Smith and Montgomerie 1991; Teather 1992; McRae et al. 1993; Leonard and Horn 1996; Dearborn 1998) and sometimes perish (Mock 1985).

Observational studies show that late hatched subordinate offspring are lighter than earlier hatched nestlings, but they invest effort in developing wings to fledge simultaneously with their older siblings. This is because parental provisioning rates to nests decrease rapidly following the fledging of the first nestlings (Mainwaring et al. 2010b; Zach 1982; Nilsson and Svensson 1996; Lago et al. 2000). Other studies show that later hatched nestlings allocate resources to body mass and tarsus growth and away from wings (Johnson et al. 2003; Mainwaring et al., 2009, 2010a). They probably do this to maintain height within nests because nestling height in nests correlates with begging success (Leonard and Horn 1996; Dearborn 1998; Dickens et al. 2008). Alternatively, the maintenance of body mass may help nestlings from becoming a “tasty” chick because ectoparasites are found most on smaller offspring (Simon et al. 2003).

Subordinate and dominant offspring have different patterns of resource allocation during growth, with subordinate offspring investing energy in those morphological characters that help them compete with older siblings. Mainwaring et al. (2010a) examined the relative contribution of maternal and post-hatching environmental effects, which are the nestling hierarchies created by hatching asynchrony in determining those patterns in blue tits by disrupting the order in which eggs hatched so that in experimental broods, nestlings from first-laid eggs hatched last. If maternal effects were important then the experimental nestlings would grow similarly to early hatched nestlings, but if environmental effects created by hatching asynchrony were important, then the experimental nestlings would grow similarly to late-hatched nestlings. Experimental nestlings grew similarly to late-hatched nestlings, thereby showing that the environmental effects created by hatching asynchrony and not maternal effects determined growth allocation patterns (Mainwaring et al. 2010a).

In those species with sexual size dimorphism among offspring, the offspring of the smaller sex are subordinate and may thus employ patterns of differential growth to compete with the larger sex. In blue tits, female nestlings are roughly 10% lighter than males, and allocate resources to develop flight feathers (Mainwaring et al. 2011a), presumably to fledge at the same time as their male siblings. In Eurasian sparrowhawks, a species that exhibits reversed sexual size dimorphism, smaller male nestlings preferentially developed the growth of their outermost primary feathers because it enabled them to fledge at the same time as their female siblings (Moss 1979). Interestingly, there is no evidence that smaller nestlings allocate resources to growing larger gapes (Clark 1995) and instead, they preferentially grow their wing feathers.

Extra-pair avian offspring are often larger than within pair siblings and this was traditionally attributed to genetic factors, as proposed by the good genes effect (Griffith et al. 2002). However, recent empirical studies have shown that extra-pair offspring hatch earlier than within-pair offspring, and are therefore larger due to hatching asynchrony (Magrath et al. 2009; Ferree et al. 2010). Further, extra-pair tree swallow offspring had longer wings than within-pair siblings, while having comparable mass and tarsus length, thus demonstrating that extra-pair offspring growth patterns are not determined by the good genes effect (O'Brien and Dawson 2007). However, a paucity of research examining these patterns means that further research is needed to improve the understanding of the importance of the patterns outlined above in a comparative context.

Subordinate nestlings often employ patterns of differential growth that allocate resources to the development of their gapes, tarsi, or wings so that they can successfully acquire food from their parents or fledge simultaneously with their older siblings (Mainwaring and Hartley 2012). While such patterns of resource allocation are highly likely to be adaptive while they remain in the nest, diverting resources away from body mass is likely to be disadvantageous once they reach adulthood (Metcalfe and Monaghan 2001). This is because low mass at fledging is correlated with low survival and reproduction during adulthood (Kempenaers et al. 1992; Both et al. 1999) and so there may well be links to adulthood and particularly during the breeding season.

## Survival to maturity

Relatively little is known about subordinate individuals during the non-breeding season, particularly when compared to the breeding season. In part, this reflects the logistical challenges associated with following subordinate individuals throughout the long non-breeding season and in part reflects the reduced amount of aggression between conspecifics outside of the breeding season. For example, red deer (*Cervus elaphus*) stags establish dominance hierarchies through aggressive interactions during the rut when they compete amongst each other to mate with females, yet these males stay close together for the remainder of the year (Clutton-Brock et al. 1979). Despite subordinates facing less aggression from dominant conspecifics throughout the non-breeding season, they still face an uphill task to acquire sufficient food while competing with dominant conspecifics.

Competition for food is particularly intense in group-living species because of the close presence of competitive conspecifics, where dominant individuals usually acquire a disproportionate amount of food via aggressive behaviors. Hollis et al. (2004) examined how subordinate blue gouramis (*Trichogaster trichopterus*), which are tropical freshwater fish, use non-aggressive tactics to acquire food while avoiding the aggressive behaviors of dominant conspecifics. Subordinates used knowledge about the location and availability of food to employ highly effective “suboptimal” tactics to simultaneously obtain food and minimize the number of attacks they received from dominant conspecifics. Such tactics improved over time, thereby suggesting that subordinate fish learned to improve their chances of obtaining food (Hollis et al. 2004).

In cold regions of the northern hemisphere, subordinate individuals and species struggle to compete with dominants for food (Lahti 1998). Social hierarchies in non-breeding season flocks of birds are perhaps most notably studied in the *Parus* genus that compete for food during the cold winter months and they have provided insights into how subordinate individuals and species attempt to acquire food (Lahti 1998). For example, subordinate blue tits were excluded from the safest sites and were thus forced to forage at less safe, open sites containing less vegetative cover than dominants, where Eurasian sparrowhawk attacks on blue tits were more successful (Hinsley et al. 1995). At the interspecific level, meanwhile, dominant willow tits (*Poecile montanus*) and crested tits (*Lophophanes cristatus*) mainly fed at favored sites in trees, while smaller and subordinate coal tits (*Periparus ater*) and goldcrests (*Regulus regulus*) were mainly relegated to feed in sites on the outer branches, where the risk of predation was much greater (Suhonen et al. 1992).

While subordinate individuals and species within the *Parus* genus struggle to obtain food, the same principles may not be present in gregarious species (Gomes et al. 2022). The social networks of wild-caught common waxbills (*Estrilda astrild*) were quantified across two non-breeding seasons and years, and those subordinate individuals were found on the periphery of groups in both years. These findings demonstrate that the position of individuals within groups was repeatable and also that the social phenotype of the group was stable. Further, the position that individuals occupied with groups was not influenced by measures of their personality (Gomes et al. 2022).

## Reproductive success

We have a good understanding of differential growth in birds, yet the consequences of such resource allocation patterns remain poorly understood. This is almost certainly an oversight because early life conditions are known to influence performance during adulthood (Royle et al. 2002b; Birkhead et al., 1999, 2006; Blount et al. 2003; Stamps 2006; Criscuolo et al. 2008; Naguib et al. 2008; Mainwaring and Hartley 2013; Spagopoulou et al. 2020; Crosland et al. 2022). This is likely caused by the logistical problems with monitoring subordinate individuals after fledging, which is exacerbated by their low post-fledging survival rates. This is because subordinate birds that fledge with low body masses suffer high mortality rates and reduced mating success during adulthood (Both et al. 1999). Body size is an important determinant of breeding success in socially monogamous blue tits as smaller males acquire significantly lower fertilization success in terms of within-pair and extra-pair copulations (Kempenaers et al. 1992).

Arnold et al. (2007) examined the link between diet quality during early life and morphology during adulthood. Captive zebra finch (*Taeniopygia guttata*) nestlings were raised on either a control-quality diet of mixed seeds and conditioning food or a low-quality diet of pearly white millet. The control-quality diet had significantly higher amounts of nutrients in the form of protein, carotenoids, vitamin, E and vitamin A. Nestlings raised on a low-quality diet were lighter and had shorter tarsi during growth than nestlings on control-quality diets (Arnold et al. 2007). That pattern was, however, reversed when those birds on a low-quality diet were switched to a better diet because they caught up with their mass growth and beak color development, but not with tarsus length, when compared to birds on a high-quality diet. Females on low-quality diets had significantly shorter wings, presumably as a direct consequence of allocating resources into sex-specific structures instead of skeletal growth and feathers (Arnold et al. 2007).

Other studies of captive zebra finches provide mixed evidence of links between early life conditions and attractiveness during adulthood. Naguib et al. (2008) manipulated the size of broods in which nestlings were raised because experimentally larger broods were considered to provide worse conditions during early life than experimentally smaller broods as the individual offspring should receive less parentally provided food. However, mate choice trials showed that females did not prefer males raised in smaller broods to males raised in larger broods, despite males from smaller broods singing more than males raised in larger broods (Naguib et al. 2008). While this study provided no evidence of male attractiveness varying at the between-brood level, Mainwaring et al. (2012) used mate choice trials to show that late-hatched males within asynchronously hatched broods were more attractive to females during adulthood than early hatched males (Mainwaring et al. 2012).

Despite these insights, links between early life conditions and performance during adulthood are poorly known. This is particularly true in long-lived species because of the time and financial commitments needed to acquire information. Nevertheless, wild rhesus macaques (*Macaca mulatta*) that experienced hurricanes during early life bred at a younger age than conspecifics that never experienced hurricanes, but then exhibited a higher reproductive output during their prime reproductive years, meaning that subordinate individuals born during hurricane years overcame such early life adversity (Luevano et al. 2022).

Early life conditions also influenced the reproductive success of wild boar with poorly fed juveniles going on to have lower body masses as adults and lower levels of reproductive success (Vetter et al. 2016). Further, female wild boar parents with subordinate personality traits, including a reduced tendency to explore and being less aggressive, had lower levels of juvenile mortality, thereby demonstrating that timid females raised larger litters than more aggressive and exploratory females (Vetter et al. 2016).

Studies of invertebrates show that subordinate burying beetle (*Nicrophorus quadripunctatus*) males are competitively inferior to larger males during contests over access to females, who also actively rejected copulation attempts by smaller males (Suzuki 2009). However, Pascoal et al. (2018) showed that subordinate male burying beetles (*Nicrophorus vespilloides*) stimulate greater female fecundity than dominant males. Female beetles copulate with several different males which induces competition between rival males, with males benefitting by producing competitive sperm and by stimulating females to increase the number of eggs they lay and thus, their fecundity. Smaller, subordinate, males were better able to stimulate female fecundity than were larger, dominant, males and by inducing females to lay more eggs, subordinate males caused each larva to be smaller because of a trade-off between offspring size and number. Females thereby produce smaller larvae, even when sired by dominant males, thus providing evidence of telegony (Pascoal et al. 2018).

In group living mammals, males physically compete to establish dominance hierarchies and thus access to females. In red deer, dominance hierarchies among males are well established via non-aggressive posturing, with male reproductive success being highly skewed toward dominant males. Subordinate males do not fight dominant males because fighting results in up to 6% of rutting males being injured each year, meaning that subordinates are best off not challenging dominant males over access to females (Clutton-Brock et al. 1979; Clutton-Brock and Vincent 1991).

The situation is somewhat similar in soay sheep (*Ovis aries*), although Preston et al. (2001) showed that subordinate males acquire paternities late in the rutting period. Females copulate with seven or more different males during their 2 day fertile period, known as oestrus, and males fiercely compete for access to females during that time by physically fighting with each other. Larger males with longer horns were dominant over smaller conspecifics with shorter horns, and larger males sired more lambs during the early stages of the rut. However, the dominant males that were copulating up to 13 times per day became severely depleted of sperm, so that subordinate males produced more ejaculates than dominant males toward the end of the rut, and thus went on to sire more lambs during that period (Preston et al. 2001).

In cooperatively breeding mammals, meanwhile, dominant individuals sire offspring that are helped by subordinate helpers. Subordinate individuals are strongly constrained from siring offspring themselves by the dominant individuals. Although subordinates accrue indirect fitness benefits through raising related offspring, Young et al. (2007) examined whether subordinates may sire offspring during forays into the territories of other neighboring groups and demonstrated that subordinate male meerkats (*Suricata suricatta*) conduct regular extra-territorial forays during periods of peak female fertility. They often copulated with females in other groups and genetic analyses revealed that extra-group paternity accrued, while prospecting contributed substantially to the breeding success of subordinate males. In fact, it was the main way in which subordinate males sired offspring, with 70% of their offspring being the result of such extra-pair forays. This led to subordinate individuals significantly reducing their age of first reproduction, because they did not have to wait to become dominant within their groups and allowed them to breed without dispersing to permanently live in a new group. Estimates suggested that extra-group forays result in individuals siring 20–25% of all young in the population and in this way, subordinate meerkat males use alternative reproductive tactics to sire many offspring (Young et al. 2007).

In cooperatively breeding bird species, meanwhile, studies show that a shortage of breeding opportunities means that subordinate individuals who are unable to successfully compete for a territory instead remain on their parent’s territory and help them raise their offspring (Woolfenden and Fitzpatrick 1977; Hannon et al. 1987; Nelson-Flower et al. 2013). Within cooperatively breeding species, there, subordinate individuals have considerably lower levels of reproductive success. In turn, many studies show that subordinate individuals exhibit elevated glucocorticoid secretion in response to the stress suffered while losing competitive interactions to become a breeder (Creel 2001). Irrespective of the exact mechanism, subordinate individuals suffer disproportionally as seen in a study of acorn woodpeckers (*Melanerpes formicivorus*) where subordinate individuals suffered higher mortality rates than dominant conspecifics following a poor acorn crop (Hannon et al. 1987).

In cooperatively breeding Southern pied babblers (*Turdoides bicolor*), subordinate females rarely reproduce themselves. This means that they attempt to aggressively compete for breeding opportunities with dominant females and in the process of doing so, reduce the breeding success of dominant females (Nelson-Flower et al. 2013). Although subordinate females were unable to outcompete dominant group members, it does nonetheless highlight that although most research has focused on males, females also deserve attention. Dominance hierarchies in females have been studies in cooperatively breeding species as well as lekking species.

Lekking bird species are those that reproduce on leks, where males display in clustered groups, and females select males based on their physical position within the group and/or their plumage characteristics (Earl et al. 2022b). In Indian peafowl (*Pavo cristatus*), females with darker ornamentation and fewer circulating hormones were socially subordinate to females with brighter plumage and more circulating hormones, suggesting that female ornamentation is a reliable indicator of dominance (2022; Earl et al. 2022ab). In lance-tailed manakins (*Chiroxiphia lanceolata*), female dominance was related to preen oil volatile compounds, although dominant females acquired the preferred males (Whittaker et al. 2019).

In mammals, female topi antelopes (*Damaliscus lunatus*) compete for males at the center of leks and subordinate females attempt to physically disrupt their dominant conspecifics in an attempt to obtain copulations with preferred males at the lek center (Bro-Jørgensen 2002). Competition between females for preferred males in lekking species is intense and subordinate females try to make the best of a bad situation by physically disrupting dominant females in order to try and obtain copulations with preferred males.

## Conclusions and further research

Subordinate individuals are those raised in broods under high levels of predation risk, a scarcity of food, and an abundance of ectoparasites or are those raised by poor-quality parents or in poor-quality territories, or, alternatively, are those that hatch or are born late within asynchronously hatched broods (Stoleson and Beissinger 1995). They are generally smaller and lighter throughout the growth period than their earlier hatched and thus older siblings, which is at least partly, because they preferentially invest resources into the growth of morphological characteristics such as enlarged gapes that help subordinate individuals obtain food and fledge simultaneously with their older siblings, respectively (Saino et al. 1998; Gil et al. 2008).

Such differential growth benefits subordinate individuals in the short term. Although it is usually assumed that such growth patterns have negative consequences during adulthood, evidence for this is largely lacking at present (Mainwaring and Hartley 2012). Further research could therefore usefully examine the long-term consequences of differential growth in subordinate individuals. Such individuals are usually expected to have lower survival rates after leaving the nest, yet this has not been proven. Further, such individuals are usually lighter than dominant individuals and it would be interesting to examine if they can attain the same mass as dominant conspecifics during adulthood.

During the breeding season, subordinate individuals often fail to attract a mate or, in socially monogamous species, are cuckolded by dominant individuals (Griffith et al. 2002) and so have low levels of reproductive success. Subordinate individuals do, however, attempt to maximize their chances of acquiring copulations via a number of strategies. For example, they allocate resources into being sexually attractive during early adulthood (Mainwaring et al. 2012), disperse to new areas to attempt to acquire copulations (Young et al. 2007), or take advantage of those periods of time when dominant conspecifics have depleted their energy reserves to acquire copulations themselves (Preston et al. 2001). Subordinate individuals therefore appear to take advantage of situations in which they may otherwise lose out on mating opportunities to maximize their own reproductive success.

During the non-breeding season, meanwhile, subordinate individuals must survive to the following breeding season. This is challenging because they are often forced to roost in cooler locations than dominant conspecifics and heterospecifics so that they lose more energy during the cold winter nights (Lahti 1998). Nevertheless, subordinate individuals show adaptive behaviors to acquire food while avoiding aggressive dominant conspecifics (Hollis et al. 2004). Subordinate individuals struggle to compete against dominant individuals but have evolved to use “suboptimal” behaviors to maximize their chances of acquiring food and reproducing successfully (Hinsley et al. 1995). This means that we have acquired a good understanding of the ways in which subordinate individuals attempt to overcome the disadvantageous circumstances they encounter, although there are areas that are worthy of further research attention for furthering our understanding of the relationships between early development and later social dominance.

Subordinate individuals are known to use several “suboptimal” tactics and behaviors to overcome the disadvantageous circumstances they find themselves in. It is likely that such tactics, including foraging in ways that minimize contact with dominant conspecifics and making forays into neighboring territories to acquire copulations, represent ways in which subordinate individuals optimize the options they have available to them, yet this is unclear. Further research could therefore usefully examine whether such tactics are indeed used because subordinate individuals would fail if they directly competed with more dominate individuals and so given their competitive limitations, such tactics are actually the best options they have access to. From a game theory perspective, subordinate individuals may be choosing not to pursue the best options available to them because there is a high likelihood they would not be able to succeed in that choice, and this topic is worthy of further attention.

Subordinate individuals experience adverse conditions during early life, and although it is widely assumed that early life adversity is linked to low survival rates and low levels of reproductive success during adulthood, such links are largely lacking. Although studies of birds, such as zebra finches, raised in captivity have followed individually marked birds from their birth through to adulthood (Mainwaring et al. 2012), longitudinal studies of individually marked birds, and other taxa, in the wild would be informative in this regard.

Pertinently, studies that follow individually marked individuals throughout the entirety of their lives would enable us to examine temporal trade-offs in performance to examine possible trade-offs between performance and survival in subordinate individuals. In this regard, studying the “tactics” and behaviors that subordinate individuals use to maximize their reproductive success over the course of their lifetimes would enable scientists to examine temporal patterns of subordination and dominance. This is important as it has been shown that subordinate individuals take advantage of those periods of time when dominant individuals have depleted their energy reserves to acquire reproductive success (Preston et al. 2001), and searching for similar patterns in other species would be worthwhile.

Greater use of tracking technologies should enable us to acquire a better understanding of links between early life and adulthood. This is particularly relevant for smaller species because the development of ever-smaller tracking devices has enabled increasingly small species to be tracked. This will enable scientists to increase the number of species that can be tracked, thereby increasing the phylogenetic diversity of species for which we have data. Further, studies of individually marked animals would also enable us to examine the role of “personality” in determining the fitness of subordinate individuals. This is important because although it may appear that submissive and quieter individuals are at a disadvantage, it may not always be the case.

Further research should examine how climate change may influence links between early life conditions and performance during adulthood. Temperatures during early life influence the metabolic rates (Stawski and Geiser 2020), survival rates (Nord and Nilsson 2016), behavior (Stawski and Geiser 2020), and reproductive success (Albon et al. 1987) of individuals during adulthood and so research examining how increasing temperatures alter the performance of subordinates would be useful. Environmental conditions were found to be much more important than the size of marine colonial bryozoan (*Watersipora subtorquata*) offspring in determining their longevity as adults (Lange and Marshall 2016), thus highlighting the importance of environmental conditions in determining the performance of subordinate individuals.

## Notes

From the symposium “Pathways to adulthood: environmental, developmental, and evolutionary influences on the ontogeny of form and function” presented at the annual meeting of the Society for Integrative and Comparative Biology virtual annual meeting, January 3-7, 2023.
